# Comments on “Size dependence of the lattice parameters of carbon supported platinum nanoparticles: X-ray diffraction analysis and theoretical considerations,” *RSC Adv.*, 2014, **4**, 35959–35965

**DOI:** 10.1039/d1ra05649b

**Published:** 2022-03-08

**Authors:** Erhard W. Rothe

**Affiliations:** Department of Chemical Engineering and Materials Science, Wayne State University Detroit MI 48202 USA erothe@wayne.edu

## Abstract

Leontyev and colleagues presented the results of an experiment and of its theoretical consequences. The interpretations were based on model-fits to that experiment. Unfortunately, they used two demonstrably incorrect parameters in their models. When the correct parameters are used, the best fits, and the corresponding theoretical implications, are interchanged. Specifically, they deduced an inapplicability of the Laplace–Young equation to the compression of nanoparticles. After their faulty parameters are corrected, this is no longer proven. An equation based on Laplace–Young pressure was dismissed by Leontyev *et al.*, but when recalculated with corrected parameters, it fits their experimental data points.

## Abstract 2

Leontyev and colleagues presented the results of an experiment and of its theoretical consequences.

## Introduction

**Fig. 1:**
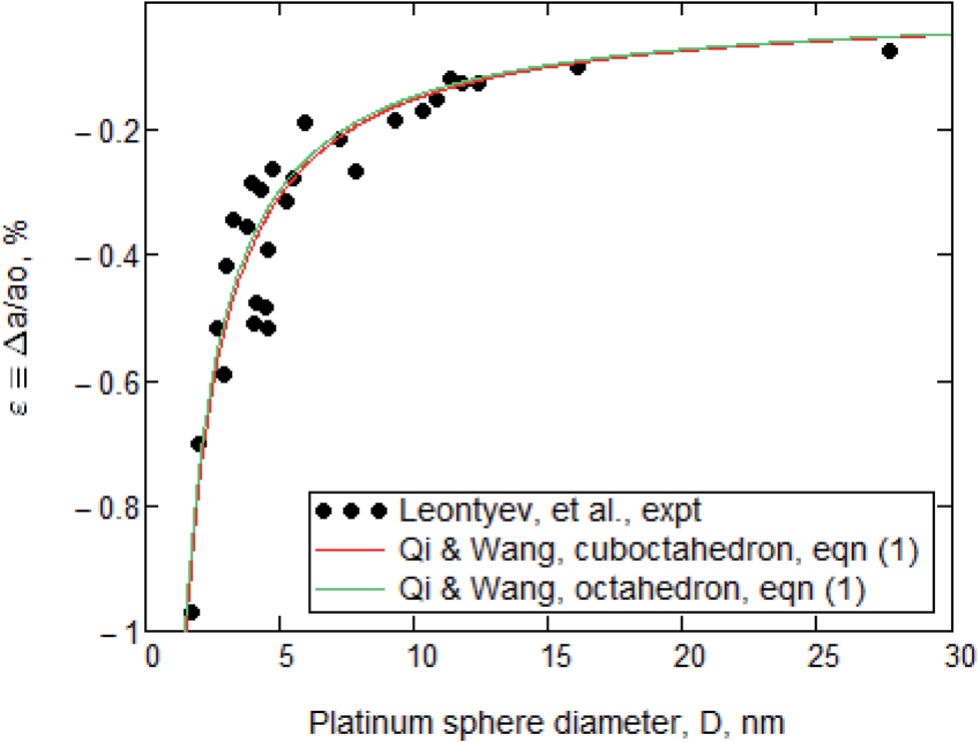
Normalized plot of platinum lattice parameter as a function of particle diameter, *i.e.*, *ε*(*D*). The data points are taken from Leontyev. They *appear* to be well fitted by the Qi and Wang model, *i.e.*, eqn (1), as shown by the red and green lines. That good fit was the basis for Leontyev's claim for its suitability, but that pertains only when the *incorrect* value of *G* is used. Fig. 2 will show the result of using the correct *G*.

**Fig. 2:**
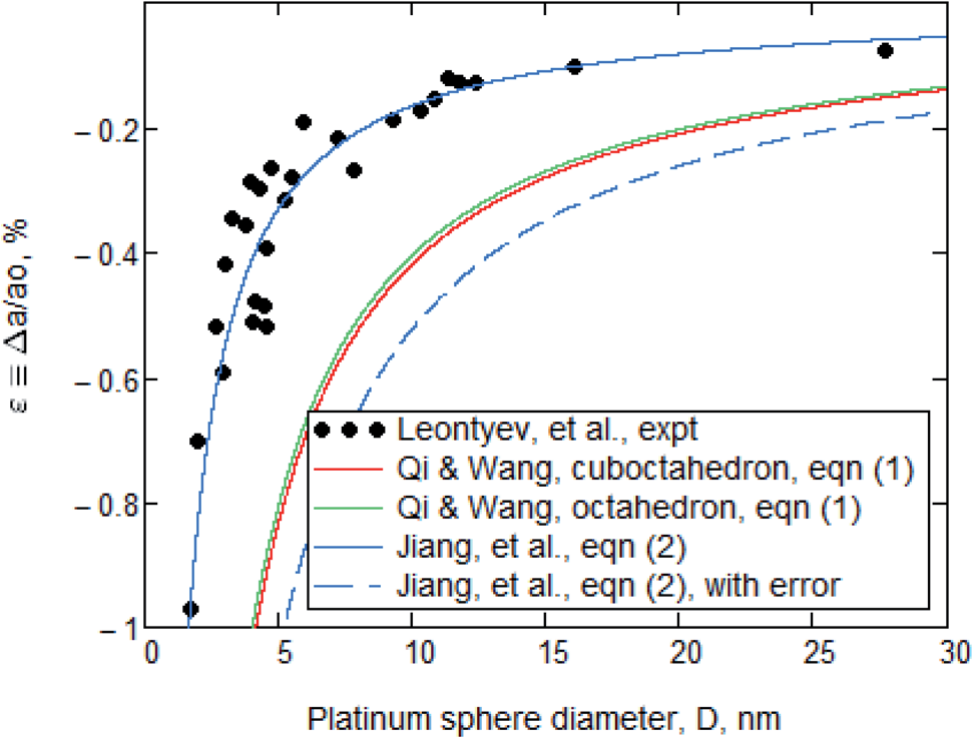
Normalized plot of platinum lattice parameter *versus* particle diameter. The data points are the same as in Fig. 1. The red and green lines are obtained using eqn (1) with a correct value for *G*: *i.e.*, 61.9 GPa. That recalculation served to lower their position with respect to the data points. The solid blue line is from eqn (2) with parameters that are tabulated in ref. 3 and it is a good representation of the experimental points. The dashed blue line represents Leontyev's version of eqn (2), but with a 10× too large value for atomic diameter *h*, and it was presented by them as evidence that eqn (2) was unsatisfactory.

Leontyev *et al.*^1^ measured the effect of particle size upon the lattice parameter of nanocrystalline platinum. They reported the determination of *a*(*D*), where *a* is the lattice parameter and *D* is the particle diameter, in a range 3 nm < *D* < 27 nm. Those experimental data are displayed in both Fig. 1 and 2 and we have no reason to doubt them.

Unfortunately, in the interpretation of these experimental data, they employed two incorrect physical properties of platinum in their models. Therefore, their conclusions will be shown to be invalid.

## Identification of the two errors

Both errors probably resulted from look-up failures from data compilations.

An analysis involving eqn (1) below requires a value for the shear modulus (aka, the modulus of rigidity) *G*. They used *G* = 168 GPa, but it is actually ≈ 62 GPa. Table 1 shows five sources for this assertion, as well as values for Young's modulus. The latter are included only to suggest that Leontyev, *et al.* probably took Young's modulus from a compilation, but misattributed that value to be for *G*.

**Table 1:** Values for platinum's shear modulus (aka modulus of rigidity) *G*, Young's modulus, and atomic diameter *h*. Leontyev's use of *G* = 168 GPa and of *h* = 2.78 nm is wrong and leads to the incorrect conclusions discussed in the text

	Used by Leontyev, *et al.*^a^	Compilation 1^b^	Compilation 2^c^	Merker, *et al.*^d^	Farraro and McLellan^e^	Darling^f^	Compilation 3^g^
Shear modulus, *G* (GPa)	168	61	60.9	54.2	62	62.2	
Young's modulus (GPa)		168	170	164.6	159	174	
Atomic diameter, *h* (nm)	2.78	0.272	0.26			0.2774	0.2775

a I. N. Leontyev, A. B. Kuriganova, N. G. Leontyev, A. Rakhmatullin, N. V. Smirnova and V. Dmitriev, *RSC Adv*., 2014, **4**, 35959–35965.

b https://www.webelements.com/platinum/physics.html, accessed July 2021.

c https://environmentalchemistry.com/yogi/periodic/Pt.html#Physical, accessed July 2021.

d J. Merker, D. Lupton, M. Topfer and H. Knake, *Platin. Met. Rev*., 2001, **45**, 74–82.

e R. Farraro and R. B. McLellan, *Metall. Trans. A*, 1977, **8**, 1563–1565.

f A. S. Darling, *Platin. Met. Rev.*, 1966, **10**, 14–19.

g H. W. King, in *Physical Metallurgy*, ed. R. W. Cahn, North-Holland, Amsterdam, 1970, p. 60.

Another analysis, see eqn (2) below, requires a value for the atomic diameter *h*. Table 1 shows that they used a value that is 10× too large. That error might have been caused by a mix-up between nm and Å units.

## The effect of the shear modulus error

Fig. 1 contains the experimental data points and Leontyev's best fit to them. Those points were taken from Leontyev's Fig. 2, which is a plot of *a*(*D*). Our Fig. 1 is a normalized version, *i.e.*, of *ε*(*D*), where *ε* ≡ Δ*a*/*a*_0_, Δ*a* ≡ (*a* − *a*_0_) and *a* → *a*_0_ when *D* → ∞.

They reported that the best fit to their experimental data was a “continuous-medium” approach of Qi and Wang,^2^ who had derived the equation [Leontyev's eqn (4)]1
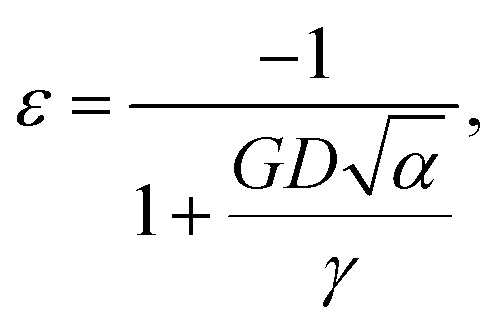
where *α* is a nanoparticle shape factor, which is unity for a sphere, and *γ* is the surface energy. Fig. 1 contains a red line and a green line, both of which were generated from eqn (1) with the parameters used by Leontyev. Those for the red line were *α* = 1.105 (cuboctahedron), *G* = 168 GPa, and *γ* = 2.734 J m^−2^, while those for the green line were *α* = 1.183 (octahedron), *G* = 168 GPa, and *γ* = 2.734 J m^−2^. These two barely distinguishable lines display excellent, *but misleading*, agreement with the data.

However, as seen in Table 1, Leontyev's value of *G* = 168 GPa is wrong. We recalculated the red and green lines obtained from eqn (1) but using *G* = 61.9 GPa instead. As shown in Fig. 2, the red and green lines now differ substantially from the data.

## The effect of the atomic diameter error

Also shown in Fig. 2 is a solid blue line that does agree with data points. It was generated from eqn (2) [Leontyev's eqn (6)], which was derived by Jiang, *et al.*^3^ This is2
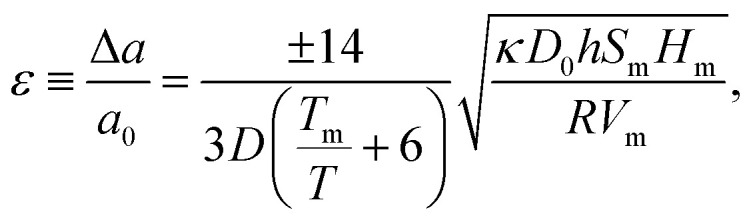
where the parameters are the ideal gas constant *R*, the melting temperature *T*_m_, the molar melting enthalpy *H*_m_, the compressibility *κ*, the molar melting entropy *S*_m_, the molar volume *V*_m_, the atomic diameter *h*, and *D*_0_ = 3*h*. The solid blue line was calculated with parameters that are tabulated in Jiang's^3^ paper.

In contrast, Leontyev, *et al.* also reported that the results from eqn (2) did not fit the data: they generated, instead, the dashed blue line. But their dashed blue line was calculated with the erroneous 10× larger value for *h*. Accordingly, their value for |*ε*| is √10 greater.

They do not specify what value they used for *D*_0_. If, as reported by Jiang,^3^ who derived eqn (2), it is 3*h*, there should have been a further factor of √10.

## Summary of the error effects

Subsequent quotation marks will indicate quotes from Leontyev, *et al.* Their *Conclusion* section states that “The comparison of the calculated dependencies based on the above models with the experimental data, shows that the results provided by the Continuous-Medium model is in better agreement than those obtained by others approaches. It is thus the best approach to simulate the unit cell parameter dependence.” The data and fits shown in Fig. 2 show that this is not correct. Eqn (2) yields a far better fit than eqn (1) when proper parameters are used, which is just the reverse of Leontyev's statement.

## The significance of the error

Eqn (2) was “based on the notion that the nanoparticles are compressed by the Laplace pressure. The value for the pressure difference of a spherical surface was formulated in 1805 independently by Thomas Young and Pierre Simon de Laplace.” That is correct. But their statement that “Laplace pressure can be confronted with various problems” and their quoted^4^ viewpoint that “the Laplace pressure is a purely mathematical concept and cannot cause compression of bodies” are no longer supported by our revised analysis.

After correcting for the apparent look-up failures, there is no basis in the data fits to suggest that the Laplace pressure concept is wrong. Instead, the excellent fit of Leontyev's data to the Laplace based eqn (2) suggests, although it does not prove, the contrary.

## Conflicts of interest

There are no conflicts to declare.

## Supplementary Material
